# The Hospital. Nursing Section

**Published:** 1904-04-23

**Authors:** 


### The Hospital.

**Nursing Section.**

Contributions for this Section of "The Hospital" should be addressed to the Editor, "The Hospital" Nursing Section, 28 & 29 Southampton Street, Strand, London, W.C.

No. 917.—Vol. XXXVI. *SATURDAY, APRIL* 23, 1904.

### Notes on News from the Nursing World.

### THE HEAD NURSE OF KING EDWARD THE SEVENTH'S HOSPITAL.

The London branch of Osborne House, to be known as King Edward the Seventh's Hospital, which was opened for the reception of officers of both Services on Monday at 9 Grosvenor Gardens, is in charge of Miss Agnes de Keyser, the founder, who is henceforth to be known as Sister Agnes. The head nurse is Miss Fletcher, who, with Miss Haines, now the matron at Osborne House, attended the King during his illness in 1902. Excellent accommodation has been provided in the hospital for the nursing staff.

### QUEEN ALEXANDRA'S MILITARY NURSING SERVICE.

We are officially informed by the Matron-in-Chief of Queen Alexandra's Imperial Military Nursing Service, that Miss M. E. Richardson, staff nurse, has been posted to the Royal Herbert Hospital, Woolwich, and Miss L. M. Moor, staff nurse, has been posted to the Royal Arsenal, Woolwich; Sister Caroline Hutton Potts has also been promoted to the rank of matron; Miss M. C. S. Knox, R.R.C., matron, has been transferred from Cambridge Hospital, Aldershot, to the Western Heights, Dover; Miss G. E. Saunder, matron, from the troopship *Plassy* to Portsmouth; and Miss A. E. Tait, matron, from the troopship *Plassy* to York; Miss E. C. Stewart, sister, has been transferred from the troopship *Plassy* to Cambridge Hospital, Aldershot; and among staff nurses the following transfers have taken place:—Miss E. M. Bickerdike, from the troopship *Plassy* to the Royal Herbert Hospital, Woolwich; Miss A. F. Byers, from the Royal Herbert Hospital, Woolwich, to the Royal Arsenal, Woolwich; Miss A. E. Fitzgerald, from Woolwich to Cambridge Hospital, Aldershot; Miss M. L. Harris, from Portsmouth to the Royal Herbert Hospital, Woolwich; and Miss A. A. Wilson, from the Royal Arsenal, Woolwich, to the Royal Herbert Hospital, Woolwich.

### NURSING RUSSIAN SOLDIERS IN A BRITISH HOSPITAL.

The first authentic news of the nursing of soldiers engaged in the war between Russia and Japan comes to us from a correspondent in Hong Kong, whose communication in another column will be read with much interest. The patients whom our correspondent describes were Russians wounded at Chemulpo, and it is gratifying to learn that, although there is naturally much sympathy entertained in Hong Kong for the Japanese, this has not for a moment been allowed to interfere with the help freely accorded to the sufferers on the other side. It is also pleasing to learn that the wounded Russians evinced the utmost gratitude for the care and kindness bestowed upon them in the British Hospital. They have been afforded practical proof of the fact that the ministrations of nurses, all the world over, are gladly given on the one condition that there is need for them.

### THE COLONIAL NURSING ASSOCIATION.

The annual meeting of the Colonial Nursing Association will take place on Wednesday, June 8, at Sunderland House, Curzon Street, the residence of the Duke of Marlborough, Under-Secretary for the Colonies. Lord Grey will preside, and Princess Henry of Battenberg has consented to be present. We hope to be able to announce that the meeting will be addressed by Mr. Lyttelton, who, as Colonial Secretary, has not yet given his blessing to a movement which owes so much to his predecessor.

### LORD LINDLEY ON THE MIDWIVES ACT.

In moving the adoption of the annual report of the Norfolk District and Cottage Nursing Federation, Lord Lindley said that the greatest expense the Federation had to incur was in connection with the training of nurses, and that unless the subscriptions and donations came in more liberally in the future, they would not be able to increase the number of nurses in training. The Midwives Act, he proceeded to say, would, on coming into force, make it imperative for all midwives to be properly certified, and it therefore foreshadowed a possible difficulty to the Federation, inasmuch as if their nurses were sufficiently trained to obtain a midwife's certificate from the Central Board they might think themselves above cottage work. This, Lord Lindley urged, is a danger that will have to be faced. Meanwhile, it appears that the Federation has suffered from the prevalence of an impression that, because the late and present secretaries are clergy of the Church of England, neither training nor assistance is given to persons not belonging to the Church. As a matter of fact, several of the nurses are not members of the Church of England, and their services are freely given to all classes of the community irrespective of their religious denomination.

### THE MATRON OF STOCKPORT HOSPITAL CENSURED.

An inquiry, lasting from three o'clock on Monday afternoon to one o'clock on Tuesday morning, was held by the sub-committee of the Stockport Corporation, into charges brought against the management by a nurse who has been dismissed on the grounds of insubordination. The nurse in question had been requested to attend the investigation, but she refused to be present as the committee declined to allow her solicitor to accompany her. In the course of the proceedings, the matron, two doctors, and other persons, were questioned, and the matron admitted that infected clothing had been permitted to remain in one of the wards, but she denied most of the allegations of Nurse Griffin, one of which was that practical joking had been going on with the cognisance of the authorities. A resolution, proposing that the matron should be censured, was met by an amendment that she should be asked to resign, and the resolution was carried.

### THE TRAINED NURSES' ANNUITY FUND.

As the last report of the Trained Nurses' Annuity Fund shows very clearly, that organisation is in a far from flourishing condition, several nurses who qualified for annuities under its rules a long time ago being still waiting for their income. With the idea of exciting interest and obtaining help a meeting will be held at 19 Stratford Place, the residence of Emily Lady Ampthill, on Wednesday next at 3.30 p.m. Lady Loch will preside, and the speakers will include Mrs. Garrett Anderson, M.D., Lady Helen Monro-Ferguson, Miss Barton (matron of Chelsea Infirmary), and Miss C. J. Wood.

### NEWPORT GUARDIANS AND THEIR CHARGE NURSE.

A remarkable state of affairs is described in another column by a correspondent in Newport, Monmouthshire. It appears that on Saturday the Newport Guardians considered a number of applications for the position of charge nurse at the workhouse hospital, and it was decided to appoint one of the three candidates selected by the Visiting Committee. But no sooner was the candidate informed of her appointment than she intimated that she declined to serve, and another candidate being then chosen, she also refused to take up the duties. In neither case was any adequate explanation given, and both the Guardians and the public in the thriving town of Newport are naturally concerned that two strangers should have cast what they doubtless consider a stigma on the institution under their control. As the hospital, which stands in a very pleasant position, is practically a new building, on up-to-date lines, and the quarters of the nurses are quite comfortable, we are not surprised that there is a keen desire to ascertain the real reason why the Guardians eventually had to proceed to the election of the third selected candidate, who accepted the post.

### THE NURSING QUESTION AT A DIOCESAN SYNOD.

At the Salisbury Diocesan Synod last week, Canon Sir James Philipps moved the appointment of a committee to report upon the provision made in the diocese for the care of the sick, either in hospitals or by nursing associations, especially in view of the Midwives Act; also to give counsel to Churchmen as to the channels into which their generosity in this respect might be directed. In the course of his speech, Sir Jame said that the object of the Association, recently started in Wiltshire, was not to pay for nurses, but to help in the training of them. As to the Midwives Act, at the present moment any woman of good character who had acted as a midwife for two years could be registered on the payment of half-a-guinea. But in 1910 things were to be altered, and no woman would be able to call herself a midwife unless she was certificated. He thought that unless they bestirred themselves, in six years there would be very few midwives in the country, for it was calculated that out of 500 at present in Wiltshire only about 100 would obtain a certificate. The resolution, which was supported by Earl Nelson, was unanimously agreed to.

### NORTHERN WORKHOUSE NURSING ASSOCIATION.

Two questions of much interest are touched upon in the annual report of the Northern Workhouse Nursing Association, whose staff at the end of 1903 consisted of 81 superintendents and nurses and nine probationers in training. The committee, while expressing satisfaction that the three years' training, or an equivalent training and experience, required by the Local Government Board, is being carried out more and more stringently by Boards of Guardians, think that in estimating the value of a nurse's three years' training, the size of the hospital in which she has been trained, and consequent possible amount of experience attainable under such conditions, should be considered more than it is. Their opinion is, "that a nurse trained for two years, or even for one year, in a large infirmary with every up-to-date appliance is, after an additional experience of two or three years, far better qualified for the post of either charge or superintendent nurse than is a nurse who has received her training for the full three years in one of the smaller infirmaries." No doubt this may be so, but as the period of training at nearly all the large workhouse infirmaries is now three years, there might be a practical difficulty in carrying into effect the views of the Association. We agree entirely with the committee as to the great necessity for an improvement in the style and qualifications of masters and matrons. In order to emphasise this view, the report quotes "an extreme but actual case" as follows:—"Can trained and educated nurses be expected to work satisfactorily under a master and matron, who, originally pauper inmates, rose to be porter and cook, and were finally promoted to be master and matron?" Of course, they cannot. Such persons, while fully entitled to credit for improving their own position, must be deficient in the kind of training which should be essential to the occupation of the offices in question.

### PREFERENTIAL NURSING.

At a meeting of the Norwich Board of Guardians, Mr. T. Brooks, the vice-chairman, alluded to the improvements which have been made in the nursing of the sick since he joined the Board. There are now 10 nurses for 123 people, whereas when he became a Guardian there were only two, the rest being pauper assistants. Mr. Brooks added that he was glad that pauper nursing was a thing of the past, because it was preferential. In other words, "persons visiting their friends used to hand money to the pauper nurses, who, as a consequence, would give preference to certain patients." We have no doubt that the allegation of Mr. Brooks is accurate. Unfortunately, however, although pauper assistants have been abolished at Norwich, they are still at work in other places, and with them possibly the demoralising and degrading system of preferential nursing. The use of the word nursing is, of course, a misnomer in such cases, but the worst of it is that the ignorant poor who are tended by bribed assistants, or left uncared for when their friends are unable to expend money in bribes, are sometimes led to suppose that the services of nurses can only be commanded by similar means.

### RAISING SALARIES AT BRENTFORD.

The Brentford Guardians have very wisely adopted a recommendation of the Infirmary Committee to appreciably increase the salaries of their nurses. In future, unless the Local Government Board should withhold assent—which is extremely unlikely—the commencing salary of a sister or charge nurse will be £30 instead of £28, and the maximum salary £36 instead of £34. Ward nurses will commence with £22 instead of £20, and rise by annual increments to £28 instead of £25. The new scale is to apply to the nurses at present in the service of the Board. It need hardly be said that there is nothing unusual in the augmentation. The Brentford Guardians have simply decided to come into line with other similar institutions which for some time have paid the same salaries as they have just sanctioned.

### NORTHAMPTON GUARDIANS AND THE NURSING INSTITUTE.

The Northampton Guardians have decided to make a substantial increase to their subscription to the nursing institute. The question was brought before the board by Mr. R. Hill, who stated that 2,242 patients receiving parish help were visited by the nurses of the institute last year, and having warmly eulogised the work done under its auspices, he said that he had intended to ask the Guardians to convert their contribution of 40 guineas into one of £80, which was the estimated cost of a district nurse; "but seeing all the circumstances he reduced it to 60 guineas." As another member of the board expressed a doubt whether they should increase expenditure, we conclude that the original proposal would have excited a certain measure of opposition. The resolution in favour of the augmentation to 60 guineas was carried unanimously.

### THIRTY-SEVEN MILES FROM A RAILWAY STATION.

In a modest manner the "Irish Homestead" Nursing Association, which have just issued their second annual report, seem to be doing excellent work. The object in view is to provide nursing aid amongst the sick poor of the West of Ireland or elsewhere, as funds and circumstances permit. The work at present is in the district of Kilcommon Erris, county Mayo, which is particularly wild and desolate, and extends over 80 square miles of country. It consists almost exclusively of scattered cabins, is 37 miles from the nearest railway station, and contains few even of the conveniences of the small Irish country towns. As may be imagined, the difficulties of the nurse are many, and when a vacancy occurred last July, it was not found possible to obtain a successor until early in the present year. Now a Queen's Nurse is in charge, and to provide as far as possible for her comfort, the Association have rented a cottage from the Congested Districts Board, and furnished it. Practically, the work of the Association only differs from that of Lady Dudley's organisation in the fact that it is locally administered. In the six months ending June 30th, nearly 300 visits were paid to 33 patients, and it is hoped that in future this average will be maintained. The Association are, happily, not in any financial trouble. The balance in hand at the end of 1903 was £124, but this, of course, would have been far less if the nursing had proceeded without a break.

### A WARNING TO MATRONS OF PRIVATE NURSING INSTITUTIONS.

The matron of a private nursing institution in Lancashire complains of an experience which we are glad to think is as unusual as it is unjustifiable. According to her statement, a few months ago a certain nurse joined her staff and signed the customary agreement that she would not nurse within a radius of ten miles after leaving the institution. She was sent by the matron to a case which proved to be long and remunerative. After nursing the patient for some time she severed her connection with the institution, telling the matron that she was leaving to start private nursing on her own account in a neighbouring city. The matron, naturally believing her statement, gave her a testimonial. She subsequently learnt that the nurse had never gone out of the prohibited radius, but had simply taken over for her own benefit the case to which she had been sent. The matron wrote threatening legal proceedings and demanding the return of the testimonial, in consequence of which the patient temporarily quitted the town, taking the nurse with him. As the case may come into Court, we make no comment, except to warn matrons of private nursing institutions to qualify testimonials to members of their staff by the addition that they are given on condition that no nursing work is undertaken within the limits of the original agreement. This, at any rate, would simplify matters in the event of legal proceedings.

### A LAST TRIBUTE TO A POPULAR NURSE.

The remains of Miss Clara Puddy, who was fo[illegible] many years parochial nurse in the parish of St[illegible] George's, Brandon Hill, Bristol, were laid to rest las[illegible] week amid many manifestations of regret. Mis[illegible] Puddy died suddenly from heart disease, and in a[illegible] address at the funeral service the Rev. K. M. Pi[illegible] dwelt on the faithful and loving manner in which right up to the day of her death, she had carried ou[illegible] her duties. A number of floral emblems were sen[illegible] by relatives and friends, and one of the mournin[illegible] carriages contained the Clifton and district nurses.

### ROYAL BRITISH NURSES' ASSOCIATION.

The fifteenth conversazione of the Royal Brit[illegible] Nurses' Association will be held at the Portm[illegible] Rooms, Baker Street, on Tuesday next, at 8 P[illegible] Princess Christian has signified her intention to [illegible] present. Nurses are requested to wear indo[illegible] uniform.

### The Nursing Outlook.

"From magnanimity, all fear above;

From nobler recompense, above applause,

Which owes to man's short outlook all its charm."

### HOMES—WANTED, A NEW DEPARTURE.

Amongst the numerous pamphlets that are constantly distributed, nurses receive many advertisements of homes in which different forms of cure are carried out. There are homes for rest cures, and for the fresh-air cure, and for cure by vegetarian diet and so on; and most of these homes are worked by nurses. Two things are specially striking about these advertisements: first, that the terms are very high—often as much as five guineas a week; and secondly, that the establishments are nearly all for women. The conclusion to be drawn is that the rich idle woman frequently becomes neurotic; she leads an unnatural and over-stimulated life, and has every now and then to undergo a course of treatment in order to reduce her to a passable state of health once more.

At one time it was always the "rest" cure and the hysterical girl was put to bed and kept there and fed until her nerves had quieted down and her body could take on flesh again. But now we have Dr. Bornstein declaring that in cases of emaciation and neurasthenia he would dispense with "isolation, nurses, hydrotherapy, mechanotherapy and electrical treatment"—all these are unnecessary and only meat diet and iron and quinine need be insisted upon. Then we have Dr. Haig on the contrary advising vegetarianism as a cure for most evils, and at Slough there is a "home where patients are received for the purpose of practical instruction in the uric acid free diet." Also, to take a third example before we turn to the practical point we wish to bring forward, in Hampshire there is a "Nature Cure Establishment," where there are air-baths, sun-baths, water-baths, rain-baths, and so on; where the patients walk about in sandals, sleep in windowless huts, and ever wear an air of most superior virtue. Now on the whole we believe that most of the ladies who haunt these homes would secure more permanent health for themselves, and be of some use to the world at large, if they would combine a little wholesome work for children and thought for others with their own selfish and self-centred cures.

What is badly wanted is some house in the country under the direction of a nurse, where ladies could go for two or three months, or longer, and where the simplest of lives should be the rule, but where chops or stockings would be allowed on the doctor's orders. In connection with this house there should be an annexe for sick children—poor little incurable or convalescent waifs from the London slums—cases of hip disease and paralysis and so on. And the care and teaching of these children, the actual washing and watching of them, should be part of the duty of the ladies of the house. In fact, it would be a sort of temporary secular retreat where not only the health of the body, but the mental and moral health of the inmates would be considered. How many a woman in the old days was saved by flying from idleness to three months in a hospital as a paying probationer? That door is largely closed now, and has never been available for the mass of neurasthenic girls who are not fitted for quite such drastic remedies. But surely some nurse who has herself passed through the fire, who knows that labour is necessary to the mental salvation of many of the idle rich, that a knowledge of some of the realities of life is craved by many an over-sheltered damsel, and that if you make heroic demands you often arouse heroic response, could see her way to make these cures less selfish and more permanent.

Such a house need not be very expensive; simplicity of diet, clothing and furniture would be part of its creed; there are plenty of societies ready to pay 5s. a week to have sick children cared for under a trained nurse in a healthy spot, and the patients might at least be asked to pay a pound a week to cover their board and lodging. The institution would of course have to be under a committee on which doctors and clergy should be represented, and whilst it should be quite unsectarian it should supply some mental food—lectures not only on hygiene but on the lives of great men and women, and there should be regular hours for study.

It is esssntial to secure not only the bodily results which the purely medical homes claim, but also some of the moral results obtained by temporary seclusion in a Roman Catholic or Anglican retreat, shall we say? And some of the mental results obtained by a course of instruction such as that given at the Woodbroke Settlement which Mr. Cadbury has founded. What might be hoped from residence in such an institution would be (1) better health without becoming too self-centred, and learning to seek only your own good and to ignore the sufferings of others; (2) a knowledge of how to maintain this health by living simply, and by employing regularly one's body, mind and soul; (3) a sense of the friendly brotherhood of all those who are loyal to the demands of the ideal, gained through studying the lives of St. Francis, Tolstoy, Florence Nightingale, Marcus Aurelius, and others; (4) the extending of this sympathy received to others, especially to sick children.

Even higher results might be achieved; for when once such a home got into working order, those who came under its influence might be encouraged to form some union, such as the "Union pour l'Action Morale," which states: "We would make known in our persons the usefulness of rule, of discipline, of resignation, of renunciation. We forbid ourselves to seek after popularity. We pledge ourselves to abstain from falsehood in all its degrees. We promise deliberate resistance to the tidal waves of fashion, to the panics of the public mind, and to all forms of weakness and of fear." And this would be to secure such mental health as no mere light or air or diet treatments could achieve.

### Lectures Upon the Nursing of Mental Diseases.

By Robert Jones, M.D.Lond., B.S., F.R.C.S.Eng., M.R.C.P.Lond., Resident Physician and Superintendent of the London County Asylum, Claybury.

### LECTURE X.

The arrangements of an asylum or hospital for the insane and the discipline therein maintained are intended (1) to promote the mental recovery of the patients, when this is possible, and (2) in cases where recovery does not take place, to make their surroundings as favourable, healthy, and comfortable as may be compatible with their proper custody, safety, and due supervision.

In a hospital where persons are treated exclusively for bodily diseases, and where residence is for a limited time, there are none of those intimacies and dependent feelings which result from long-continued association, forced by the personal restrictions of the lock and key, as must arise in asylums, where nurses and patients lead to a great extent a joint life, and where the nurse becomes not only the personal attendant, but the companion of the inmates. In hospitals the patients are expected to conform to the discipline and rules of the place under pain of dismissal. In asylums the patients are expected *not* to conform, and it is only by the influence and through the persuasion and example of the nurse that patients will behave with propriety, see to their personal cleanliness and immediate wants, and consent to engage in any occupation or diversion. It is by moral suasion, and not by means of compulsion that discipline and order are maintained in an asylum, and the work of the establishment is efficiently performed, so that the asylum nurse and other officers are continuously practising what has been described as "mental therapeutics." As in hospitals so in asylums, the mental nurse has an honourable and a direct course to pursue. Her duties in an institution are generally laid down for her in a book of "Rules" which she receives upon commencing duty, and she should familiarise herself with them and act up to them. She should also make herself acquainted with the general arrangements and regulations pertaining to the establishment in which she is serving, and endeavour to carry them out in a loyal manner. The duties of a nurse upon the insane may be classified as (1) general, which is the subject of the present lecture, and (2) special, (3) personal. The two latter will be dealt with in a subsequent lecture. The mental nurse, who is assumed to be a person of intelligence, kindly disposition, and pleasant manner, has the duty in the first place of being self-respecting, careful in her conversation, neat in her attire, pleasant in demeanour, and circumspect in her conduct. She should wear no jewellery or ornaments, for there are many patients who will claim these as their own, and suspect her of appropriating them, which may lead to personal encounters, and one or other may be hurt by brooches, ear-rings, pins, or the like. She should not make a promise to a patient unless this is to be fulfilled, and when it is necessary to refuse a request no feeling is aroused if the nurse replies that the rules do not permit the request to be granted. No favouritism should be shown to any patient, for the delusions of the insane are often based upon suspicion, and a partiality may rouse special antipathy. There should be no petty jealousy among nurses in attendance, and no appearance of disagreement should be exhibited before patients, as it may prove destructive to all future efforts to control and supervise the patients under their control. The nurse can do much by acts of kindness and by the exercise of tact—that special knack of knowing what to say and do at the right time—a faculty which can always, to some extent, be acquired and cultivated, more especially when there is an earnest desire on the part of the nurse to do the best she can to soothe the restless, troubled, and afflicted spirit. "A soft answer turneth away wrath," and the duties of a mental nurse call for a pre-eminent amount of self-forgetfulness and unselfishness. In no other capacity is a man or woman brought face to face with such a collection of annoying and aggravating people as those who inhabit the wards of an asylum for the insane, and in regard to whom there is no justification for resentment, and certainly not for retaliation. It is recognised by the tactful and intelligent nurse that the patients are irresponsible and are to be pitied; also, that they have been thus reduced through disease which she hopes to be instrumental in curing.

1. As to the *general* duties the mental nurse understands that insanity is largely affected by the state of the body, and everything that tends to promote the bodily health has a direct and beneficial influence on the mental welfare. It is necessary to attend to ventilation, cleanliness, and warmth; to see that the clothing is sufficient by day and by night, that food is properly and abundantly taken, that sleep is obtained, and that regular exercise is taken in the open air. She should also be competent to administer to the needs of the sick, to apply massage, and to give electric baths. There are certain special duties in connection with bathing the insane, as also in the case of infectious disease, to which we shall refer in more detail later. The nurse should have the day-rooms and bed-rooms kept clean, tidy, well-ventilated and sufficiently warm, the bed-clothing dry and aired, and the patients should be encouraged, when possible, to keep their rooms and persons clean and neat, and this to be done in a quiet and simple manner, for occupation diverts the mind from insane ideas and assists in the treatment of sleeplessness. Occupation for the insane is a most important point, and the nurse has great power in this respect by her own example over the patient, and she becomes a means of considerable assistance in carrying out the doctor's instructions. It is not the amount of work done, nor the value of it, which is a consideration, but the fact that occupation, however simple in itself, by exercising alike the bodily and mental powers has a salutary effect upon both. It tends to quicken the senses by keeping the mind from morbid fancies, it places the nervous system in a more normal state of tension, and leaves a pleasant mental and physical reaction due to acquiring a new attainment. There is a pleasure in the contemplation of something usefully achieved and done; hence it is always desirable, when the bodily health will allow, to try and induce the patient to occupy the time usefully and engage in some work that is congenial and suitable, however simple this may be. The idle should be induced to work, but the willing must not be over-pressed. The lives of people shut up in asylums, whether patients or staff, requires diversion and relaxation, hence amusements — both indoor and outdoor — are a necessary form of treatment. The manner in which the evening is spent often has much to do with the production of sleep, and discretion in carrying out directions as to amusements and occupation will call for the exercise of thoughtful qualities. The amusement must be adapted to the physical and mental condition of the patient. If reading is permitted, the right books should be carefully chosen, and if cards are resorted to they should not be made a source of excitement, but used to obtain relief. Instructions as to the amount of outdoor amusements or exercise should be carefully carried out, and those in regard to medicines punctually and accurately observed. As to food, sufficient time should be allowed for its mastication; and plates, knives, and forks, and dishes should not be removed with undue haste. The patient should always receive his or her proper amount of nourishment, and be encouraged to behave decorously and properly at table.

The doors of all wards must be kept locked when instructions are so given, and doors leading from one part of the asylum to the other are always kept closed. The law imposes certain restrictions in regard to the letters of insane patients, and the nurse should never post these when addressed by patients to persons outside the asylum; for the medical officers in charge are under penalties in respect to the disposal of the letters of patients, and all communications should be handed to them directly or through the higher officers of the institution. The nurse must also be careful as to means which patients may make use of to escape from custody. There are legal enactments in regard to the escape of patients, which, if infringed, may lead her into much trouble, and no patient should ever be entrusted with a key by any nurse. Ladders, steps, or other means of escape must be carefully put away, as also must any article, tool, or instrument which can be made use of, either for the purpose of escape or to injure themselves or others, nor are any instruments to be used by the patients out of the nurse's sight. Nurses upon the insane should never talk with people outside of patients under their care, nor of their conduct, as such a course might cause injury to the friends of patients, and may cause the nurse to lose her post. The law is particular about the property of patients in asylums, who through being incarcerated are thus deprived of the power to manage their own affairs, and the nurse is not permitted to take charge of money or property belonging to the patient, nor to sell, to buy, nor borrow from a patient. Some institutions are very particular about nurses accepting presents or gratuities in the shape of money or gifts from the friends of patients, and it is well for the nurse always to decline these.

Nurses in asylums are, like those in hospitals, graded in rank, and the senior nurse of a ward is responsible for the stock of the ward, over which she has control and charge. She should be careful to preserve this stock from damage or loss, and from time to time she should check the stock with the inventory handed to her on first taking up her duties. It is most necessary that nurses upon the insane should be absolutely punctual on duty, and special hours are fixed for the day and night staff. The regular hours for rising, taking food, work, exercise, amusement, and retiring to bed are most beneficial, not only the bodily health, but also to the mental state of the insane, for they help them to regular life and re-educate them into good and regular habits. If the nurse is unpunctual her remissness may be the cause of loss of life through suicide, for which she must be held responsible. Moreover, her bad example will react upon the other nurses. Strict obedience and loyalty are expected from the mental nurse, but emergencies may arise in regard to any patient which the special experience and the initiative of the mental nurse will qualify her to respond to. This much has been said about her requirements in regard to those committed to her care. There are others she owes to herself, viz., care for her own health; she must have ample sleep, food, and time to take it; exercise and recreation both of body and mind, and regular holidays at definite intervals, also means to enjoy them, without which she will not be able to keep that charm of cheerfulness and resourcefulness which pre-eminently characterises the mental nurse, and which are invaluable acquirements in her work.

### Nursing Russian Soldiers Wounded at Chemulpo.

BY OUR OWN CORRESPONDENT.

Of course I gladly accepted an invitation from a Russian friend to accompany her round the wards of the Government Civil Hospital in Hong Kong, where seven of her disabled countrymen lay, only regretting that a very limited knowledge of their language prevented direct communication with them. Yet I comprehended enough to know that Madame's gifts of cigarettes, cigars, and flowers were practically superfluous, owing to the lavish generosity of the officers and crew of his Majesty's ship *Amphitrite*, the ship that conveyed them from the scene of action, the first engagement at Chemulpo.

### "Seriously Wounded."

Six of the men, including two petty officers, were rescued from the *Variag*, and were all labelled seriously wounded, nearly all having two or three wounds. One produced a jagged splinter of lead abstracted from a chest wound, and told me with delight that he was getting on "all right" (his only English words) now, for "Sisster-ah" (Sister) had discovered and removed a small piece of cloth which had prevented his wound from healing. Another had lost a huge piece of flesh from the abdomen, and the worst case, in addition to a damaged thigh and chest, had had two ounces of lead removed from his arm near the wrist, the bones and tendons being completely shattered, but the patient alleged with joy that the doctors of the hospital and the great English doctor (Sir Frederick Treves) who examined him, and also the seventh man, a lieutenant with a damaged knee cap, had said they could save the limb although they were afraid it would scarcely be useful.

### Gratitude and "Chaff."

The men were all most grateful for the care and kindness bestowed upon them from the moment of rescue, and lingered with child-like affection upon the merits of Jack Tar, with whom they parted sorrowfully. With no knowledge of any language but their own, the sister told us that they achieved a good deal of communication with patients of all nationalities in the ward, by means of dumb show, and she pointed out the Chinese "Boy" (male nurse) whom they had dubbed the Rooskie (Russian) doctor, because after some days he was allowed to do their minor dressings. They chaffingly promised him a decoration for his breast, the size of a huge brass spittoon, as soon as they got back to their country and in touch with the Czar.

### Ancient Newspapers.

We paid many visits to the wards, and distributed useful gifts, but I was forcibly reminded of Russia's prohibitory measures by the total absence of literature bearing upon a subject in which the wounded soldiers were deeply interested, viz. the war. *L'Illustration*, a French paper, copies of which dated back as far as 1898, were the most up-to-date journals provided by Madame, and a tacit silence upon current events seemed to be observed. Still, from English papers and the other patients, they had learnt a good deal, and fresh developments were eagerly looked for.

### A Gallant Officer.

The Lieutenant, in a ward by himself, is in far happier case, for he possesses a fair knowledge of English which will

**NURSING RUSSIAN SOLDIERS WOUNDED AT CHEMULPO**—*Continued.*

do much towards alleviating the tedium of a necessarily protracted rest since his knee-cap had a piece deeply scooped out of it and narrowly escaped fracture. His bravery in sticking to the guns while shell and shrapnell did their deadly work by clearing the decks around him, has already received his Czar's acknowledgments and the highest Russian decoration, that of St. George, is to be conferred upon him.

Dejected Farewell.

On the occasion of my second visit four of the six seamen were grouped, dejectedly taking leave of their fellows, whose bed of sickness perhaps seemed a happier billet than the prospect of a merciful but untried imprisonment at Colombo, but perhaps the news which has just reached me is true and they are to be returned to their mother-country on parole.

### The Registration of Nurses: Our First Debate.

BY ONE WHO WAS PRESENT.

After we had drunk our coffee, the matron got up and said that as in such a busy life as hers, she had not so much opportunity as she would wish for exchanging opinions with her sisters on the current topics of the nursing world; and that she had thought it might be for the good of us all if we met now and then to discuss subjects of interest connected with our work. She thought that the burning question of the day for nurses was certainly that of State Registration; and she would like first to hear what Sister Medical had to say on the topic.

Sister said she felt that the time was well on its way when it would be as necessary for nurses to be registered as it is now for them to be vaccinated; it was in the air, and every intelligent nurse must come to one conclusion or another on it. She thought it was a distinct step in the right direction; but she could not express her hope too strongly that the movement should not be rushed on in a hurry, believing that without grave consideration of every detail there would be a great risk of the nurse of the future being turned out a most objectionable person; a machine-made article, with all the finer qualities of a nurse left out. She hoped that legislation on the subject would shape it into something that would do the minimum of harm.

Sister Front was then asked to speak; which she did very much in favour of the proposition. She thought the objects of registration excellent; viz., the protection of the public in their search for capable nurses, and the looking after the good repute of the profession. But she wanted to know why so many representatives of the great London hospitals had ranged themselves against registration. The matron replied that she thought there was less opposition than might appear; for though many matrons, perhaps somewhat impulsively, had given their names as opponents, yet many of the leading men connected with hospitals strongly upheld the desirability of affixing some sort of hall mark to properly-trained nurses. Some doctors also were in favour of it; and no doubt more would be enlisted in the ranks of supporters as the scheme was brought more into form. Both the existing bills were in some respects impracticable; but she thought that a committee of experts would soon arrange something that could be worked. Their aim would be to equalise the training of all nurses by a process of levelling up.

Matron then called on Sister Noah to speak. Sister produced such a packet of closely-written sheets of paper from her apron pocket that we thought she could not have been so busy as some of us had been all day. She argued that the nurse of the present day had developed from the status of Mrs. Gamp quite as far as was necessary for herself or her patients; that if she were enrolled in a State register after passing a State examination which could only take cognisance of her acquirements without any test of her real fitness for the work, she would lose what little was left to her of the sense of the work being a vocation and would become a hard, professional, unsympathetic sort of tradeswoman. Indeed, she did not see in what respect the Society of Registered Nurses as it was foreshadowed would differ from a trades union. She thought, too, that the entire value of a certificate depended on the person who grants it; and that a testimonial signed by a matron standing at the head of her profession would outweigh any State-granted parchment. She suggested that if something had to be registered, it should be the training schools and not the individual nurses, then the public would know they were getting what they asked for, viz.—a thoroughly taught nurse, and also they would have the nurse hall-marked by the one person who had a real knowledge of the way in which the nurse had acquitted herself for at least three years. Indeed, there is no knowing how long Sister Noah would have gone on if little coughs and a prolonged yawn from one of her hearers had not cut her short.

There was a considerable pause next, while everyone was thinking that it was nearly time to give the report to the night nurses. However, some sister opened the ball again by asking the matron what sort of examination it was proposed to establish. How could it be sufficiently practical? What was to happen to the excellent nurses one came across frequently, who could make their patients as comfortable as possible, and could keep them in good humour, and without worrying them knew exactly from hour to hour what progress they were making, or what bad symptoms were developing; but who could not pass the simplest written or *vivâ voce* examination to save their lives? Were they to be left outside the ranks of hospital nurses for the future? The matron answered that the examination would be entirely one to prove education and capability; the suitability and character of the nurse being left, as heretofore, to the matron in whose school she had trained, and whose aim it would be to weed out the unfit as soon as possible. She thought that it was necessary to move with the times, and that uneducated women would find it increasingly difficult to get appointments, though no doubt it might be years before there were enough women capable of registration to fill all the posts requiring trained nurses. Registration would in no way lessen the responsibility of the matrons in choosing suitable women.

Then Sister Kitchen asked Sister Noah in what way a nurse trained in a registered school would be different from a registered nurse; but by that time we were nearly all talking together and her response was lost. During a momentary lull the taking-in sister, who had only slipped in during the last 10 minutes, and who had evidently been thinking more of how to arrange another emergency bed than of the subject of the debate, asked, à la "little Peterkin," "But what is the object of State Registration?" Whereat we all laughed together, and the matron, saying that she thought it was a measure which would be entirely for the good of all trained nurses, wished us good night.

### The Training of Probationers in Small Hospitals.

A FEW weeks ago it was suggested in the columns of THE HOSPITAL that it might be well if English hospitals followed the practice, recently introduced, of some of the Chicago hospitals, and gave their probationers an opportunity of gaining experience in district nursing. As this has been done for some time past at the hospital to which I am now attached, a short account of how the system works may be of interest.

### A CHANGE OF OPINION.

It was in a large London hospital that I received my training; and when I heard girls proposing to enter some little hospital as probationers, I used to try and dissuade them, pointing out that if they ever wished to be sisters in large hospitals they must go through the training also in a large hospital. I always think the years one spends as sister in a "smart" ward of a good hospital are the happiest in one's nursing career; but as one grows older one sees that it would not do for every nurse's ambition to be the same, and that a large proportion of the present-time probationers must be contented to turn into the useful and necessary private nurse or district nurse at home or abroad.

With this end in view, I have recently come to the conclusion that no more suitable training-ground could be found for them than the small provincial hospital of 40 or 50 beds, providing the training is adapted to fit them for the particular work which they mean to take up.

I will try to briefly describe the training we hope to offer to probationers who enter for a three years' course, trusting that it may lead to some discussion on the advantages of our scheme, and possibly may help other small hospitals in framing new rules and regulations, with reference to nursing education.

### THE NEW WING.

In the new wing of the Victoria Hospital, Keighley, there are on the ground-floor two wards for men—one of 16 beds and one of four beds—also two private wards of one bed each; on the upper floor there is one female ward of 16 beds, a children's ward of eight beds, and two private wards of one bed each—48 beds in all. There is a large theatre block on the ground-floor with anæsthetic room, sterilising room, etc. The nurses home, opened in January, is large and comfortable, each nurse having a separate bedroom; there is plenty of bathroom accommodation and electric light everywhere. The nurses' dining-hall is situated in the kitchen block, and is very convenient; there also are the work-room, the store-rooms, etc.; and on the floor above are the servants' bedrooms.

### THE NURSING AND DOMESTIC STAFF.

To work this hospital our nursing staff will consist of—on day duty, matron, sister for ground-floor and theatre, sister for upper floor, two staff nurses (probationers with over two years' training) and six probationers; and on night duty a night sister and two probationers. We have recently done away with "scrubbers" entirely, and find it more economical to have plenty of resident maids, all wearing uniform. Our domestic staff consists of a laundress and her assistant, a sewing-maid—who makes the uniform for nurses and servants, besides making and mending for the wards and home—a cook, a housemaid, a dining-hall-maid, a kitchen-maid and two ward-maids; there is also a man who attends to the furnaces, cleans windows, etc., and a hall boy who attends to the telephone, messages, etc. This will probably appear rather a large domestic staff for the number of the beds, but the hospital has been built on a large scale, with a view to future expansion, so that there is a great deal of cleaning work.

### THE DISTRICT NURSE.

Besides the nurses on the regular staff, we have a fully trained district nurse living in the hospital, but maintained by the Charity Organisation Society. She works under the doctors of the town, including those on the staff of the hospital, who find it a great convenience to be able to telephone to the hospital at any time about any case they wish visited, and she also visits patients discharged from hospital on account of pressure upon the beds before their wounds are quite healed, as we have no out-patient department. Most of the patients are treated gratuitously, but if they can afford to do so they pay according to the rental value of their houses, on a scale ranging from 4d. to 1s. per visit. The district nurse is frequently called upon to help the doctors at operations. In all these duties she is assisted every morning by one of the probationers, who, while she is acting as "district probationer," assists in the children's ward from 8 to 9 A.M. and again in the evening, as a rule taking her off-duty time in the afternoon.

### PRIVATE NURSING STAFF.

We also have a private nursing staff of six or seven nurses. Most of them were trained here, and they are kept constantly employed—mainly by the doctors on the staff. They assist in the wards and theatre when in the hospital for more than a day or so. If they are all engaged, and one of the doctors requires a nurse, he is often glad to have one of our senior probationers, and it is good for them to begin private nursing under doctors who know something of their capabilities.

### THE COURSE FOR PROBATIONERS.

Briefly, the course we hope to keep our probationers to will be as follows: They come for two months on trial. The first six months at any rate will be spent as junior probationer on day duty; during the next six months they will be liable to do three months' night duty as junior probationer. During their second year they will do two months as theatre probationer, and will act as senior probationer on day or night duty—not more than three months at a time of the latter—as required. During the early part of their third year they will act for two months as district probationer, filling in the rest of their time as senior probationers or as staff nurses in the wards, or, occasionally, as private nurses. The probationers pay no premium and receive no salary the first year; they receive £10 the second year and £15 the third year. Uniform is provided after the two months' trial. When they are appointed staff nurses, or when they are acting as district probationers, or when they are sent out private nursing, they receive a little extra pay, so that the third year's pay is seldom so low as £15. There is a good fever hospital near here, and they often send to us for an extra nurse (private), and are quite willing to take our senior probationers, so that, if they wish to do so, they can thus gain a little experience in fever nursing.

### THE LECTURES AND OPERATIONS.

The doctors and matron give courses of lectures to probationers, and the probationers are expected to pass an examination before gaining their certificate. The medical and surgical cases are fairly well divided, but the following list of some of the operations performed here during the last three months will show that the surgical cases are good ones: Hernia 1 case, spina bifida 1, Cæsarian section 1, amputation of breast 3, gallstones 1, tracheotomy 1, gunshot

### THE TRAINING OF PROBATIONERS IN SMALL HOSPITALS.—*Continued.*

wound of abdomen 1, ovariotomy 4, sarcoma of axilla 1. At present, unfortunately, we cannot afford a house surgeon, but hope to be able to have one soon. The hospital is not as well supported as it might be, and moving into the new building, with the additional expenses thus entailed, is naturally causing some anxiety to those responsible for current expenses. At the same time, we are very anxious that the work of renovating the old building should be put in hand as soon as possible, as there would not be the slightest difficulty in filling the 30 or 35 additional beds that would thus be available. The house surgeon would then become a necessity, and the value of the training offered to the probationers would be largely increased.

### An Extraordinary Incident at Newport, Monmouthshire.

BY OUR OWN CORRESPONDENT.

FOR some few years past there has been a good deal of trouble at the Newport Workhouse Hospital in getting nurses either to apply for positions, or, after they have got them, to keep them. Both the Visiting Committee of the workhouse and the whole body of Guardians have either winked at the cause, or causes, or have not troubled themselves to properly investigate the whole matter to the bottom. An incident which happened on Saturday last, however, is so glaring that even if the Guardians were ever so much inclined to let it slide the general public of the union would press for an investigation either at the hands of a special committee or by direct application to the Local Goverment Board.

About a year ago it was thought that the probationers may have had some cause for complaint on the ground of want of proper and systematic instruction. To meet any such assumption as this the Guardians settled upon a plan of regular lectures by the medical officer (Dr. Macormack) and the superintendent nurse (Miss Jones). But this has not been found to get rid of the difficulties. Six weeks ago a well-educated and apparently eminently suitable young lady was given a trial, but at the end of her month declined to continue the duties, and would not tell the Guardians why she went home.

The climax came on Saturday. The Guardians having advertised in THE HOSPITAL for a charge nurse for the workhouse, received a large number of applications which the visiting committee thinned down to three, out of which the final selection was to be made. The three left in the final running were Alice Jane Haydon Pike of Weston-super-Mare, Violet Hannah Edmunds of the Union Infirmary, Romford, and Martha Ann Hodgkins of Camden Town, London, N.W. Miss Edmunds and Miss Hodgkins appeared before the Board at the offices of the Guardians without going to the workhouse; Miss Pike had apparently mistaken her instructions and had gone to the workhouse thinking that the appointment was to be made there. When the vote was taken it was found that Miss Edmunds had secured a very large majority, Miss Hodgkins was second, and Miss Pike was very far behind. But when Miss Edmunds was called before the Guardians and informed of her appointment, she at once told them that she preferred not to take the appointment as she had heard something since her arrival in the town which caused her to decline. She would not inform the Board why she changed her mind, or what had been said to her, or by whom.

Some of the Guardians were indignant. Mr. Abrahamson protested that it was not right that members of the Board should interview candidates and give them a bad impression of the staff and institution unless they had the courage of their convictions and were inclined to speak out.

Mr. W. H. Brown, the vice-chairman, said that it was a curious thing to him that Miss Edmunds, who only arrived in the town an hour or two before the appointment was made, with the hope of getting the appointment, should have changed her mind in so short a time.

Mr. W. Evans moved that the matter be referred to the Visiting Committee to investigate, also to see if there were any probationers at the hospital who were qualified to take the position.

This was not agreed to, and the Board went to a second vote with the result that Miss Hodgkins was appointed. On being informed of her choice, Miss Hodgkins also declined the position. The reason she gave was that she did not know until she arrived at Newport that there was a superintendent nurse at the hospital.

Again it was proposed that the matter be referred back to the Visiting Committee so that an investigation might be made, but the Guardians in the end decided to give Miss Pike a month's trial.

But the matter is not likely to be allowed to remain quietly where it was left by the Guardians. A new board has just been appointed. The sitting on Saturday was the first after the triennial election and there was so much business to dc that several members were too much occupied to specialise upon any particular point. There is, however, a sufficient strain of new blood on the board to take the matter up in earnest and see what the true position is. It can hardly be that the dislike to the Newport Workhouse Hospital arises from insufficient or improper quarters. The workhouse and hospital have only just been reopened after a thorough overhauling and rebuilding, entailing a cost of about £70,000.

After the meeting on Saturday, I interviewed some of the members and officials of the board.

The Chairman of the Guardians (Mr. Thomas Dutfield) said that he was quite taken by surprise when both the accepted candidates declined the appointment, and did not know what their ground of complaint was. So far as he could understand, it was simply the outcome of a bit of idle gossip.

The Medical Officer (Dr. Macormack) said that he was unaware of the cause of complaint.

Several of the Guardians were of opinion that the whole matter should be thoroughly investigated so as to place the nursing staff on a sound basis. It was pretty freely hinted that Miss Edmunds was influenced, in her refusal of the office, by what she heard from one or more of the lady Guardians.

### Presentations.

PLYMOUTH WORKHOUSE INFIRMARY.—A pleasing ceremony took place on Friday last, when Sister Hale was presented with a silver toilet set and bouquet, on her resigning her appointment after 6½ years' service. The presentation was made by the superintendent of nurses as a mark of esteem from the medical officer, the nursing staff and herself. Sister Hale has resigned owing to the serious illness of a friend.

### Everybody's Opinion.

[Correspondence on all subjects is invited, but we cannot in any way be responsible for the opinions expressed by our correspondents. No communication can be entertained if the name and address of the correspondent are not given as a guarantee of good faith, but not necessarily for publication. All correspondents should write on one side of the paper only.]

### STATE REGISTRATION.

Miss Jessie Pick, matron of Aughton Isolation Hospital, near Ormskirk, writes: I should like to add my name to the manifesto against State registration. I feel that it will do more harm than good.

### PORTRAITS AS PRESENTATIONS.

"Ward Sister" writes: Some of the sisters I know are far too modest to wish to have a portrait of themselves presented to them, but I think that they would exceedingly appreciate a miniature of a favourite doctor or surgeon under whom they had worked. The idea seems to me to be so good because it has both the charm of novelty and suggests many interesting possibilities.

### DISTRICT NURSING IN CAMBRIDGE.

Miss A. P. Wilson, Lady Superintendent of the Cambridge District Nursing Institution, writes: In thanking you for your insertion of a notice of our institution, may I draw your attention to the fact that the particulars of last year are incomplete. The visits mentioned are only for parish cases. The return for 1903 is as follows:—General cases 1,020, visits 33,082; parish cases 96, visits 5,813; maternity cases 247, visits 2,706. In addition to the six nurses referred to in The Hospital there are two maternity nurses engaged by the Institution who attend respectable poor married women for a fee of 5s., which includes attendance for 10 days.

### FILLING A WATER-BED.

Nurse Dowling writes: Having had considerable experience in one of the largest hospitals in London, I consider the most safe and best method of filling a water-bed is to fill the bed on an adjoining one and then lift the patient carefully on to it. I fail to see what benefit the patient derives from rolling the water-bed under him. I should think that it would only tend to discomfort. "M. G.", I think will admit that the essentials of nursing are not only far more thoroughly taught now, but nurses have more opportunities of learning new ways and ideas. Therefore the nurse of the present should, in my opinion, be superior and more scientific in every way than the nurse trained in the eighties.

"Matron" writes: I should like to say, *re* the subject of filling water-beds, that in a very few critical cases where, if possible, no movement is to be made, the water-bed is best drawn under the patient with the blanket, mackintosh, and sheets, all at one lifting of the body, and then filled, great care being exercised not to wet the clothes; but might I add, if the case is not too heavy, it is much easier and as effectual to partly fill the bed with air; it is also much lighter, and not so liable to be burst when moved. If you half fill the bed with water the desired warmth, and then use the bellows of an air-bed, or if not at hand, a bicycle foot-pump, with a rubber tube fixed on it, you will make the bed comfortable and the proper softness. Otherwise, in most cases it is no doubt more expeditious to prepare one bed and lift the patient from one to the other, the water-bed at the same time may have been half filled with air. Commonly, and almost without exception, nurses are trained to do the latter way, as the general run of cases requiring water-beds is not so severe, and is more of the long-standing kind, and the water-bed mostly used measures about 30 inches by 36; full lengths are rare. It is a fact that in many hospitals and infirmaries nurses never see or are taught the first way, but it is no doubt very desirable they should know both ways.

### NURSING AT QUEENSBURY ISOLATION HOSPITAL.

"The Caretaker" writes: As a twenty years' reader of your valuable journal, I take this opportunity of ventilating a grievance, which I am sure neither you nor any one who understands hospital administration would approve. I am the caretaker, my wife the nurse, of an isolation hospital at Queensbury. We are the only resident staff. The medical officer of the hospital is also medical officer of health. Unfortunately, he has no authority in the administration of the hospital. The chairman of the hospital committee is the leading magnate and nothing whatever can be done without consulting him. On February 23rd a case of scarlet fever was admitted to the hospital; another on the following day, and on the 3rd of March another. Prior to the admission on the 23rd of February there were no cases in the hospital. On this date I asked the medical officer for the services of a washerwoman. He referred me to the chairman of the hospital committee, saying that he had no power to engage any assistance for the hospital. I followed up the chairman from the 3rd to the 14th of March, when he sent us a woman; before this we were doing all the night and day nursing between us. On the 15th two more cases came in, and on the 19th two more cases—total cases seven; ages from three to ten years, all in three wards. On the 18th of March I called on the chairman for someone to assist in the nursing, as both the nurse and myself were completely worn out; he ignored any suggestion of another assistant, and after I made a little bother he sent me a woman on the 20th who is untrained and has never been in a hospital and does not understand anything about the work. I ask you, in justice to the patients as well as to us, to aid in exposing this neglect.

[We have made inquiries into this matter and the answer of the medical superintendent is that since the opening of the hospital in January, 1894, only four of the 259 patients admitted into the wards have died. This does not dispose in any way of the caretaker's most justifiable complaints. In all cases of the same kind at an isolation hospital another temporary trained nurse ought to be engaged. Moreover, the caretaker, unless he be a trained nurse in charge of male patients, should not assist in the nursing at all. We have received several complaints respecting the management of isolation hospitals, which show that inspection is urgently needed.—Ed. The Hospital.]

### Appointments.

Craigleith Hospital, Edinburgh.—Miss Evelyn M. B. Simson has been appointed charge nurse. She was trained at Barnhill Hospital, Springburn, Glasgow, and Belvidere, City of Glasgow Fever Hospital.

Royal Sea-Bathing Hospital, Margate.—Miss Adeline E. Cable has been appointed sub-matron. She was trained at the London Hospital, where she has been staff nurse, ward sister, and assistant home sister. She has also served as a member of the Army Nursing Service Reserve during 1900, as sister in the Imperial Yeomanry Hospitals in Cape Colony.

Sheffield Royal Hospital.—Miss E. Towers Minors has been appointed sister of the ophthalmic wards and theatre. She was trained at the Walsall and District Hospital, and previously she had one year's ophthalmic work at the Birmingham and Midland Eye Hospital.

Stirling District Asylum, Larbert, N.B.—Miss E. Satchwell has been appointed matron of the male department, and Miss Janet E. Thyne assistant matron. Miss Satchwell was trained in general nursing at the London Hospital and in mental nursing at the Stirling District Asylum, where she has been assistant matron and night superintendent. Miss Thyne was trained in general nursing at the Edinburgh Royal Infirmary, Edinburgh.

Tanfield Infectious Hospital, Co. Durham.—Miss E. Harron has been appointed matron. She was trained at the City of London Infirmary, where she was afterwards charge nurse. She has also been charge nurse at the North-Western Fever Hospital, Hampstead, London, and sister at Islington Infirmary, London, N.

### Echoes from the Outside World.

### Return of the King and Queen.

The King and Queen left Copenhagen on Monday morning and arrived in London on Tuesday evening, both looking extremely well. They drove in an open carriage to Buckingham Palace, and were loudly cheered all along the route. Last week they paid a visit to the co-operative Trifolium dairy at Hasley, the largest establishment of its kind in the world. The owners, about 50 in number, are chiefly great landed proprietors. There are 7,000 cows, about 300 persons are employed in the dairy, the yearly production of butter is about 1,000,000 lbs., and of cheese 1,600,000 lbs., while the daily sale of milk in Copenhagen is more than 8,000 quarts. Their Majesties stayed for an hour in the establishment, inspected all parts, and asked a good many questions. The King and Queen also inspected the telegraphone, a new invention by M. Paulsen. It is an electro-magnetic apparatus, which registers telephonic messages in the absence of a recipient, and can afterwards be made to repeat them.

### Prince and Princess of Wales at Hammersmith.

On Friday last the new school for girls at Brook Green, connected with St. Paul's School, was opened by the Prince and Princess of Wales. The school, which has cost about £70,000, has been provided by the Mercers Company, who are the trustees of the Colet Foundation. It stands on about two acres of ground on the south side of Brook Green, has a good playground at the back for hockey and kindred games, and a large hall 80 feet long by 45 wide, with classrooms opening out. There is a lecture theatre, a chemical laboratory, and a "physics" room. In addition there is a "cookery" classroom, a drawing-schoolroom, and a gymnasium. The school has started with 70 pupils, and there is accommodation for 400 girls. Miss F. R. Gray is the first headmistress. The Prince and Princess of Wales upon their arrival were saluted by a guard of honour, formed by the Volunteers belonging to St. Paul's Boys' School, and the girls sang "God Bless the Prince of Wales." The Princess declared the building open amidst much cheering, and the Prince, in a short speech, said that as a member of the Mercers Company he was especially glad to be present, and that both he and the Princess trusted that the future work of the new institution would be as vigoro us and useful as that of the old school had been throughout its long and distinguished history.

### The War in the Far East.

The most striking event so far in connection with the war between Russia and Japan has been the sinking of the flagship *Petropavlovsk*, which, under the command of Admiral Makaroff, had put to sea in pursuit of a Japanese squadron, but was obliged to return into the harbour by the reinforcement of the Japanese ships. Near the entrance to the harbour the vessel struck a submarine mine laid by the Japanese, and went down. Admiral Makaroff and some 700 men were drowned, and only 32, among whom were the Grand Duke Cyril, escaped, all of these being wounded. The news of the disaster, which was mentioned in our later editions last week, created the utmost consternation in Russia, and there have been expressions of regret and sympathy at the fate of Admiral Makaroff from all quarters, including Tokio. His loss to the Russian naval forces is said by experts to mean far more to them than the loss of his flagship. On Saturday it was officially stated that the Imperial Palace at Seoul was destroyed by the fire on the preceding Thursday evening. The Emperor of Korea sought refuge in the library of the Imperial Household Department, where he was guarded by Japanese troops. The outbreak was accidental. The issue of a Russian Note intimating that correspondents using wireless telegraphy will be treated as spies, has provoked a protest. The population of Vladivostock is in want of provisions, and many of the inhabitants are quitting the town.

### The Budget.

On Tuesday afternoon Mr. Austen Chamberlain, in the capacity of Chancellor of the Exchequer, introduced his first Budget. Amongst the members of the House of Commons present was the Chancellor's father. The following is the Budget in a nutshell. The estimated expenditure for 1904-1905 is £142,880,000 and the estimated receipts £139,060,000, showing a deficit of £3,820,000. In order to meet the deficit, Mr. Austen Chamberlain has added a penny to the income tax, which is estimated to produce £2,000,000; twopence per lb. to the tea duty, making it 8d. instead of 6d., which is estimated to produce £2,000,000; a shilling per lb. on imported cigarettes, sixpence per lb. on cigars, and threepence per lb. on shredded tobacco, which is estimated to produce £550,000. This gives a total of £4,550,000, leaving a surplus of £730,000 to be carried forward.

### Death of Dr. Samuel Smiles.

The death of Dr. Samuel Smiles, author of "Self Help," took place on Saturday at his house in Pembroke Gardens, Kensington. Dr. Smiles, who was in his 92nd year, and had spent upwards of 70 years in active intellectual and other labours, started life as a medical man in Haddington. He did not succeed, perhaps because he was the youngest of eight doctors in a town with only 300 persons, mostly in good health, and eventually, in 1837, he became editor of a Leeds paper. Marrying in 1845, he relinquished his editorship in order to become assistant secretary of the Leeds and Thirsk Railway. This appointment he continued to hold until 1854, when he was appointed secretary of the South Eastern Railway. He finally retired from railway service in 1866, but his "George Stephenson," which made his fame, and the greater work which followed, "Self Help," were published before his retirement. The latter has been translated into 17 languages, and in England alone reached a circulation of 200,000 copies. Afterwards, other works followed in quick succession, and a paralytic stroke in 1871 only temporarily interfered with his literary occupations, neither his spirits nor mental power being affected by the illness.

### Vassili Verestchagin.

The artistic world sustained a great loss in the death of the famous Russian painter, Vassili Verestchagin, who was drowned in the sinking of the *Petropavlovsk*. His talent was of a particularly realistic order, and he is sometimes called "the Tolstoi in Art." He was born in 1842, and was destined for the Imperial Navy—on board one of whose vessels he ultimately lost his life—but he left the service, almost as soon as he entered it, at the age of 17. At 19 he won a silver medal at the St. Petersburg Academy with a picture of "Ulysses Slaying the Suitors." He then went to Paris and studied under Gérôme, quitting the French capital to join Governor-General Kaufmann in his Central Asian expedition. In the campaign in Turkestan he fought so bravely before Samarkand that he obtained the Order of St. George, the highest Russian distinction. In 1873 he visited British India, and his Indian pictures are well known in England. He took an active part in the Russo-Turkish War, was severely wounded, barely escaping with his life, and was present at the engagement at the Shipka Pass. One of his most celebrated pictures—in three stages—is "All Quiet in the Shipka Pass." He endeavoured to reproduce with strict truth the horrors of war, and his pictures are so realistic and terrible that it is said when they were shown in Berlin the Emperor forbade the Guards to visit the exhibition lest they should come to regard war as disgusting instead of honourable.

### A Book and its Story.

### AN ANONYMOUS AUTOBIOGRAHY.*

"Rather in all to be resigned than blest."—*Keble.*

"THE HOUSE OF QUIET" is a book of an exceptional kind. It is the life history of a man who was compelled by ill-health to withdraw early from active life after a successful university career and a brief tenure of an official post. Having to give up all serious occupation, he betook himself to his country estate. The book is formed by extracts from diaries containing reflections on the deep things of Nature, and life, and some character sketches. It is specially interesting as bringing the reader into touch with the personality of the writer. This, as revealed, points to one in whom strong intellectuality, great reverence for religion, and a disinterested desire to devote a life of enforced leisure to the service of his immediate neighbours, were dominant characteristics. The following passage gives a glimpse of his own feeling on the subject of an autobiography:—"Whether or no the book will ever see the light I know not. . . . But if ever it comes to be published I think there are some to whom it would appeal, as the thin-drawn tremour of the violin stirs the note in a vase or glass that have stood voiceless and inanimate. . . . As a pilgrim may tell his tale of travel to homekeeping folks, so I may allow myself the license to speak and to tell of the good and evil things the world has brought me and of my faint strivings after that interior peace which can be found and enjoyed."

The author had formed a philosophy which sustained him through life, and this makes the book of value; it should be one which will console and help other sufferers in like circumstances and position. It shows clearly "the possibilities of dignity and beauty that exist in the simplest and least ambitious life" (to quote from a note on the book). In youth he had not been free from the unrest, the search for truth, for religious conviction, which is natural to those who come to the haven of assured faith after much seeking. "When I contemplate this earth, with its continents and islands, its mountains and plains, all stored with the history of life and death . . . when I realise that even this world, with all its infinite records of life is but a speck in the heavens . . . the mind of man reels before the thought; and yet all is in the mind of God. From the paralysing effect of the thought that the life of the individual, when viewed from the point of its proportion to the vast scheme of the universe, can be of no definite importance, and therefore it would be wiser to resign all effort, swim on the stream, just planning life to be as easy and as pleasant as possible before sinking into the abyss," he was saved by two vital beliefs. "First, it may be confessed came the belief in the spirit of God, the thought of inner holiness, not born from any contemplation of the world around, which seems to point to far different ideals. Yet as true and truer than the bewildering example of nature is the inner voice which speaks after the wind and the storm in the silent solitudes of the soul. That this voice exists and is heard can admit of no tangible demonstration; each must speak for himself. . . . To recognise this voice as the very voice and word of the Father to sentient souls is the inevitable result of experience and thought. . . . Then came the triumphant belief, weak at first, but taking slow shape, that the attitude of the soul to its Maker can be something more than a distant reverence, an overpowering awe, a humble worship; the belief, the certainty that it can be, as it were, a personal link—that we can indeed hold converse with God, speak with Him, call upon Him, put, to use a human phrase, our hand in His, only desiring to be led acccording to His will." After having studied carefully the systems of other teachers of humanity, with the desire "to find in the great redeemers of mankind, Buddha, Socrates, Mahomet, Confucius, Shakespeare, the secret of self-conquest, of reconciliation," the knowledge slowly dawned that "In Jesus of Galilee alone we are in the presence of something which enlightens man not from within, but from without. The other great teachers of humanity seem to have looked upon the world, and into their own hearts, and deduced from thence, by flashes of indescribable genius, some order out of the chaos, some wise and temperate scheme, but with Jesus—though I long resisted the conviction—it is different. He comes not as a man speaking by observation and thought, but as a visitant from some secret place, who knows the truth, rather than guesses at it. I need not say that His reporters, the gospel writers, had but an imperfect conception of His Majesty, of His ineffable greatness—it could not well be otherwise; the mystery rather is that with such simple views of life, such elementary conceptions of the scheme of things, they yet gave so much of the stupendous truth, and revealed Jesus in His words and acts as the Divine Man, who spoke to man, not by spiritual influences, but by the very authentic utterance of God." The diaries cover the term of the author's life, from childhood to the hour of his sudden death, which occurs shortly after his meeting with the lady whom he would have married had he lived. Throughout the book, wherever it is opened, some thought clothed in appropriate words, flashes out and arrests the reader. There is a great fascination in the style, that attracts by the impression of absolute sincerity, directing the mind of the writer. It was very near the close of his life that the following beautiful passage, when face to face with death, was written. "I lie like a boat upon a quiet tide, drifting out to sea—the sea to which we all must drift. I am thankful for my life, for all its sweetness; the shadows have gone, and it seems to me now that as though all the happiness came from God, and all the shadow was of my own making. And the strangest thing of all is that the darkest shadow has always been this very passing, which now seems to me the most natural thing in the world—indeed the only true thing." Then follows a recognition, a final grace, for the crowning gift of the love that came too late. "But yesterday I murmured over having been given, as it were, so sweet a cup to taste, and then having the cup dashed from my lips. To-day I see that Love was the crown of my poor life and I thank God for putting it into my hand as His last and best gift." . . . The last recorded words are brave and beautiful ones. Space does not permit of a fuller quotation. "I have lived my little life—and my heart goes out to all of every tribe and nation under the sun who are still in the body. I would tell them with my last breath that there is comfort to the end—that there is nothing worth fretting over or being heavy-hearted about; and that the Father's arm is strong and that His heart is very wide." The lighter side of the book we have left untouched, but it has one, which is full of interest.

* "The House of Quiet." An Autobiography. Edited by J. S. (John Murray. 8s. net.)

### Notes and Queries.

**FOR REGULATIONS SEE PAGE 12.**

*Davos Platz.*

(21) I should be glad of information respecting the Sanatorium at Davos Platz. Is the new home open yet? I know several consumptive patients who wish to go.—*F. G. H.*

Write to the Hon. Secretary, the Davos Dorf Invalids' Home, 33 Curzon Street, W.

*Certificate of Housekeeping.*

(22) I would like to know where I could obtain a certificate for housekeeping without going as housekeeper into an institution?—*Sister.*

Miss Ella Pycroft, 116 St. Martin's Lane, W.C., could advise you as to institutions under the London County Council; and Miss Alice R. James provides courses of training and grants certificates in housewifery at the School of Housewifery and Domestic Science, Northfield, 101 to 106 Stamford Hill, London, N.

*Notice of Registration.*

(23) I have been registered as a midwife under the Midwives Act. Will you kindly tell me if it is necessary to send a notice to the local supervising authorities that I am going to practise before April 1st, 1905?—*R. F. C.*

You must send in a notice when you intend to begin to practise, and a like notice every subsequent January as long as you continue to do so.

*Experience.*

(24) How can a certificated monthly nurse obtain work with a midwife by which she can extend her experience?—*Miss P.*

Advertise your wish, and make private arrangement with a midwife in good practice.

*Return Fare.*

(25) Two nurses, after being in attendance on a gentleman patient for six months, resign whilst abroad: is he liable for the expenses of their return journey?—*Nurse M. H.*

It depends upon the terms of their engagement.

*Nursing in India.*

(26) Can you give me the address of any society employing nurses preferably in northern India?—*Nurse D.*

Mrs. Sheppard, 10 Chester Place, Regent's Park, N., will answer inquiries respecting the Up-country Nursing Association which supplies English-trained nurses to Europeans in India.

*Government Hospital.*

(27) I have been trained for three years, and am most anxious to obtain a post in a Government hospital, if possible abroad. I shall be very grateful if you will give me any information upon the subject.—*C. A. P.*

If you wish to become an Army nurse, apply for particulars to the Matron-in-Chief, Queen Alexandra's Imperial Military Nursing Service, 68 Victoria Street, S.W.; if a Colonial nurse, to the Secretary, Colonial Nursing Association, Imperial Institute, S.W.

*Midwife under the Poor Law.*

(28) I should be much obliged if you will tell me how to obtain a post as midwife under the Poor Law.—*L. Y.*

By advertising yourself, or by answering advertisements.

*After-Care Association.*

(29) Can you tell me if there is an association which helps to provide homes for patients well enough to leave the lunatic asylums, but who are not able to be received into their homes?—*Talie.*

The After-Care Association for Poor Persons Discharged Recovered from Asylums for the Insane, Church House, Dean's Yard, Westminster, S.W.

**Standard Nursing Manuals.**

"The Nursing Profession: How and Where to Train." 2s. net; 2s. 4d. post free.

"Nursing: Its Theory and Practice." By Percy Lewis, M.D. 3s. 6d. post free.

"Surgical Ward Work and Nursing." By Dr. A. Miles. 3s. 6d. net; 3s. 10d. post free.

"Elementary Physiology for Nurses." By C. F. Marshall, M.D., B.Sc., F.R.C.S. 2s. post free.

"Nurses' Dictionary of Medical Terms and Nursing Treatment." By Honnor Morten. 2s. post free.

"Fevers and Infectious Diseases." By W. Harding, M.D. 1s. post free.

"Art of Massage." By Mrs. Creighton Hale. 6s. post free.

### For Reading to the Sick.

"BE WITH ME LORD."

Come to me, Lord, when first I wake,
As the faint lights of morning break.
Bid purest thoughts within me rise
Like crystal dewdrops to the skies.

Come to me in the evening shade,
And if my heart from Thee have strayed,
Oh! bring it back, and from afar
Smile on me like Thine evening star.

Come to me through life's varied way,
And when its pulses cease to play,
Then, Saviour! bid me come to Thee,
That where Thou art, Thy child may be.

Strive to be as a little child who, while its mother holds its hand, goes on fearlessly, and is not disturbed because it stumbles and trips in its weakness. So long as God holds you up by the will and determination to serve Him with which He inspires you, go on boldly and do not be frightened at your little checks and falls, so long as you can throw yourself into His arms in trusting love. Go there with an open, joyful heart as often as possible; if not always joyful, at least go with a brave and faithful heart.—*S. Francis de Sales.*

The Will of God is always best. God asks nothing but acceptance of that Will, not to do great things, but what He wills. This is the true love of God in practice; this is peace in all troubles. This precludes all undue sadness or regrets, and softens all trials. It does not make one shut one's eyes, or relax one's zeal; but it preserves one's calmness in the midst of the greatest trials. It is, perhaps, the best grace God gives us. Before death, when all fades away in one's mind, one can still think of that; it is the last and brightest flicker of the flame which is going out. To accept His Will with our last breath, our last sigh, that is true love to the end, the entry into Heaven.—*Anon.*

Act faithfully according to thy degree of light, and what God giveth thee to see; and thou shalt see more clearly. Hearken to the low whispers of His voice within thee, and thou shalt hear more distinctly. Above all, do not stifle any motions of conscience. Meditate daily on the things of Eternity; and, by the grace of God, do something daily which thou wouldest wish to have done when that day cometh. Above all things, in all things, "look unto Jesus, the Author and Finisher of thy faith."—*E. B. P.*

Cherish thankfulness with prayer. St. Paul gives us in two words this secret of peace. "In everything," (he excepts nothing, so do not you), "by prayer and supplication with thanksgiving let your requests be made known unto God. And the peace of God which passeth all understanding shall keep your hearts and minds through Christ Jesus." He does not say it as a benediction only: He tells us, it "shall keep your hearts and minds." Do the one and God will do the other. Ask what you will, be thankful; and not peace only, but peace which passeth all which our poor minds can think, shall keep these poor breaking, restless hearts in Christ Jesus.—*E. B. P.*

What then? For all my sins, his pardoning grace;
For all my wants and woes, His loving-kindness;
For darkest hours the shining of God's Face,
And Christ's own Hand to lead me in my blindness.

What then? A shadowy valley low and dim,
And then a deep and darkly rolling river,
And then a flood of light, a seraph's hymn,
And God's own smile for ever and for ever.

